# Development of Nanotechnological Approaches to Improving the Antimalarial Potential of Natural Substances

**DOI:** 10.3390/molecules30204133

**Published:** 2025-10-20

**Authors:** Yoana Yoncheva, Lyubomira Radeva, Krassimira Yoncheva

**Affiliations:** 1 Faculty of Medicine, Medical University of Sofia, 1431 Sofia, Bulgaria; yyoncheva4@gmail.com; 2 Faculty of Pharmacy, Medical University of Sofia, 1000 Sofia, Bulgaria; l.radeva@pharmfac.mu-sofia.bg

**Keywords:** malaria, natural antimalarial drugs, nanoencapsulation, metal nanoparticles

## Abstract

Malaria is one of the diseases that is a serious threat to global health, with millions of cases reported worldwide in recent years. The treatment of malaria is still a challenge due to its complex pathogenesis, resistance to many of the antimalarial drugs, and adverse toxicity. Nowadays, the possibilities of applying new natural molecules alone or in combination is being researched. However, many of these substances possess low aqueous solubility, which limits their bioavailability. The solubility of such substances could be improved by applying various techniques for their nanoencapsulation, e.g., incorporation in nanocapsules, liposomes, lipid nanoparticles, etc. The current review emphasizes studies on the nanoencapsulation of some of the well-known natural antimalarial agents (quinine, artemisinin) as well as substances with newly demonstrated antimalarial potential (piperine, quercetin, etc.). The review also discusses the opportunity to simultaneously load two natural agents in nanoparticles. Special focus is given to the metal nanoparticles (e.g., silver, gold, etc.) obtained by green synthesis from plants.

## 1. Introduction

Malaria is a life-threatening disease caused by parasites that are transmitted to humans through the bites of infected female *Anopheles mosquitoes*. The parasites that affect humans include five species of the genus *Plasmodium*: *P. falciparum*, *P. vivax*, *P. ovale*, *P. malariae*, and *P. knowlesi*. Among them, *P. falciparum* is the most virulent species, causing a severe form of malaria. *P. vivax* usually causes a milder disease but has the widest geographic distribution of all the species, occurring most frequently in Southeast Asia and parts of South America. According to research, malaria, along with tuberculosis and HIV, is one of the three diseases presenting a serious threat to global health [1]. There were 263 million cases reported and 597,000 deaths in 2023 [2].

**Figure 1:**
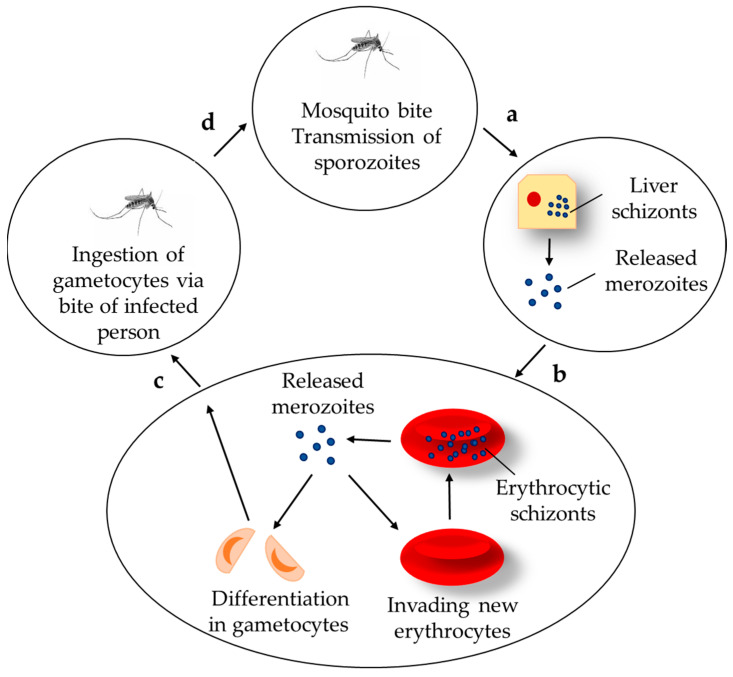
Life cycle of the *Plasmodium*: the inoculated sporozoites infect liver cells and mature into schizonts (a), the released merozoites infect the erythrocytes (b), circulation of the gametocytes in the blood stream and infecting mosquitos (c), formation of zygote in the gut and sporozoites in the salivary glands of the infected mosquito (d).

The complex life cycle of the *Plasmodium* includes stages that occur both in the intermediate host (human), as well as in the definitive host (mosquito) (Figure 1). The parasite is spread through mosquito bites in one of its forms, called sporozoites. Sporozoites then enter the liver, where they start to asexually reproduce, transforming into merozoites. In 10–15 days they are released from the liver into the bloodstream, where they start to invade erythrocytes. The parasite decomposes hemoglobin to small peptides in its digestive vacuole and releases free heme (FeII protoporphyrin IX). Since heme is toxic to the parasite, it has developed mechanisms to tackle its toxicity by dimerizing the heme to hematin and eventually forming submicronic and insoluble hemozoine crystals. The end of the erythrocytic stage is marked by the destruction of the infected erythrocytes and thus the release of the new merozoites into the bloodstream, where they are capable of infecting new erythrocytes. The frequency of the erythrocytic stages is determined by the type of *Plasmodium* causing the infection. *P. knowlesi* completes one erythrocytic cycle in 24 h, *P. vivax*, *P. falciparum,* and *P. ovale* in 48 h, and *P. malariae* in 72 h. Ultimately, some of the merozoites differentiate into a new parasitic form—the gametocyte, which is then ingested by the mosquito. In the gut of the mosquito, the male and female gametocytes form a zygote, which determines the mosquito as the determinative host, the one that facilitates the sexual reproduction of the parasite.

**Figure 2:**
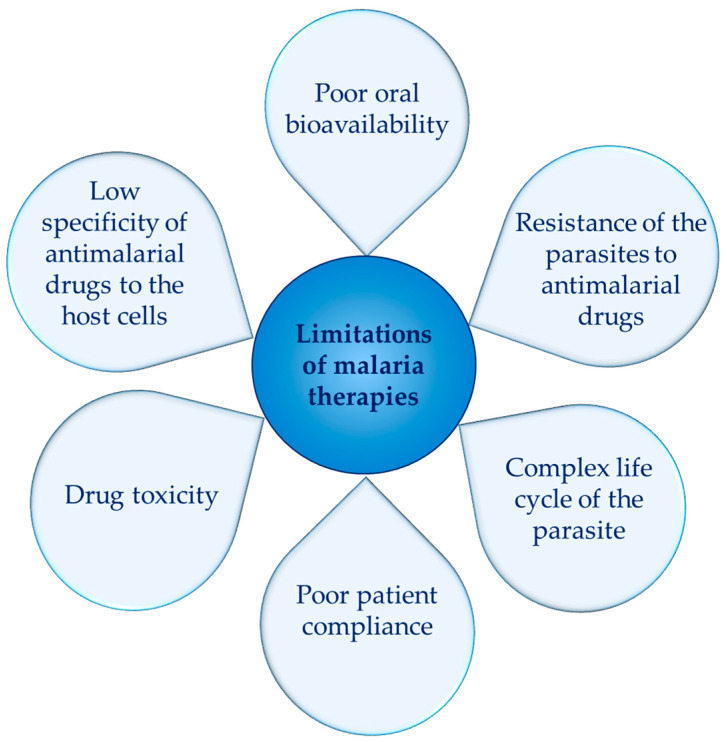
Main challenges of current antimalarial therapies.

The current antimalarial therapies are characterized by a number of limitations (Figure 2). Many of the existing drugs exhibit poor solubility, which results in low absorption and poor bioavailability. Furthermore, some malaria parasites have developed resistance to the commonly used antimalarial drugs. However, the administration of higher doses would increase toxicity, which for some groups of antimalarial drugs is inherently high. Drug toxicity is also related to the lack of selectivity of antimalarial drugs to the infected erythrocytes. Therefore, the discovery of new approaches to overcome these limitations has become urgent. Among the most researched strategies are the synthesis of new antimalarial molecules (1) and the development of innovative drug delivery systems capable of improving the bioavailability and pharmacokinetics of the existing drugs (2).

**Figure 3:**
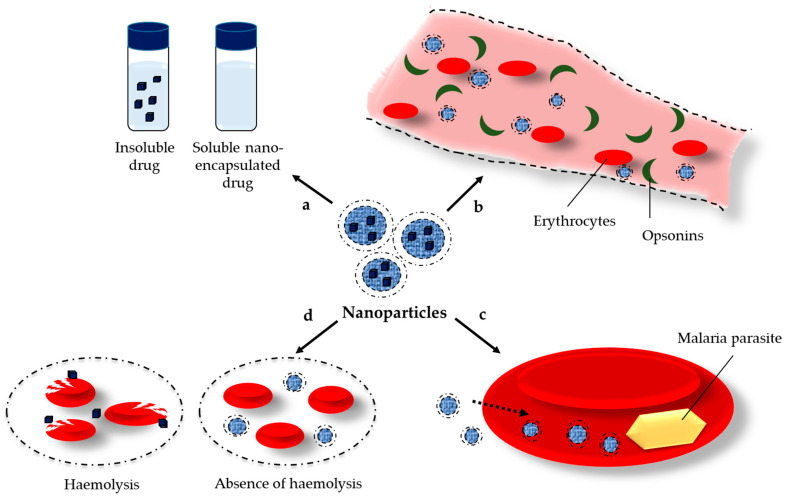
Main advantages of drug loaded nanoparticles in the treatment of malaria: enhanced aqueous solubility of the antimalarial drug (a); prolonged blood circulation (e.g., by pegylated nanoparticles) and close contact with erythrocytes (b); high intra-erythrocytic drug concentration (c); and reduced hemolysis (d).

**Figure 4:**
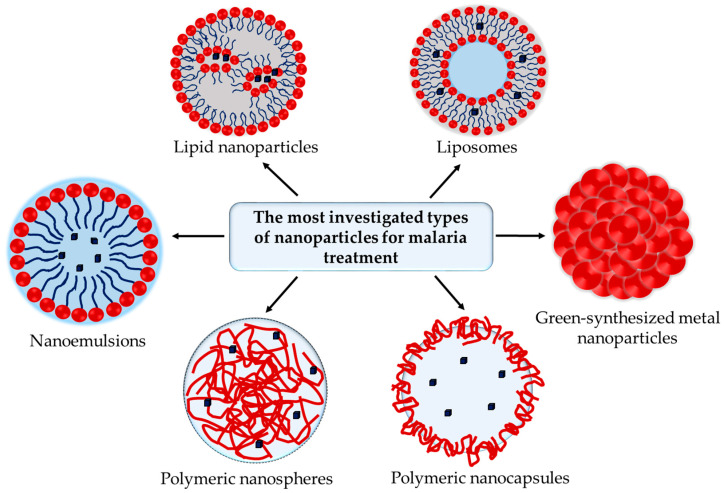
The most investigated types of nanoparticles for malaria therapies.

Nanotechnology is a powerful strategy capable of solving some of the issues of current antimalarial drugs. The nanoparticles could improve the solubility of poorly soluble drugs by different mechanisms like amorphization, micellar solubilization, etc., (Figure 3a). The surface modification of nanoparticles, especially by attachment of polyethylene glycols, avoids opsonization and provides long circulation in the bloodstream that further enables the interaction of antimalarial drugs with the infected erythrocytes (Figure 3b). Nanoparticles could ensure drug delivery exclusively in the infected host cells, minimizing its systemic toxic effects, and could ameliorate patient adherence to the therapy (Figure 3c). In addition, the high intra-erythrocytic concentration of the drugs could overcome the problem with the resistance of the parasites. Last but not least, the nanoparticles provide sustained release of the loaded drugs that could reduce the hemolytic effect of some of the antimalarial drugs (e.g., artemisinin, quinine, etc.) (Figure 3d). The nanoparticles can be formulated with a variety of available carriers, e.g., polymers, lipids, etc., or directly prepared by green synthesis from plant extracts [3]. Some of the most-researched types of nanoparticles for malaria treatment are schematically presented in Figure 4. In addition, it is important to point out that some of the carriers could exert a positive effect in animal malaria models themselves [4,5,6].

**Figure 5:**
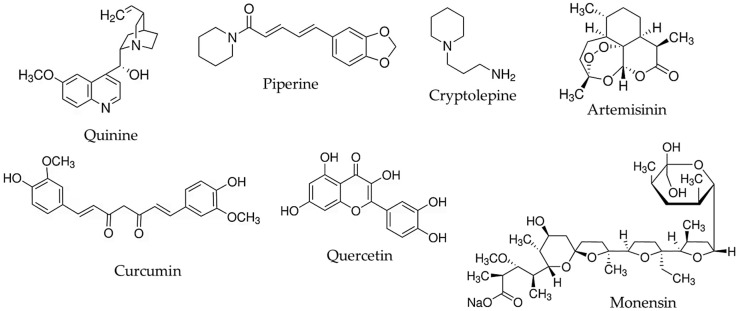
Chemical structures of the active molecules discussed in the review.

The current review discusses recent nanotechnological approaches to enhance the antimalarial activity of natural molecules. Examples for the nanoencapsulation of some of the well-known natural antimalarial agents (quinine, artemisinin) as well as substances with newly demonstrated antimalarial potential (piperine, quercetin, etc.) are summarized (Figure 5). Metal nanoparticles obtained by green synthesis from plant extracts (e.g., silver, gold, and zinc oxide nanoparticles) are also noticed.

## 2. Alkaloids

The alkaloids with antimalarial activity are divided into different classes, namely bisbenzylisoquinoline, naphtylisoquinoline, tetrahydroquinoline, furoquinoline, indoloquinoline, indole alkaloids, etc. [7,8]. Despite the structural diversity and promising antimalarial activity, the main problem of some of the alkaloids is their high toxicity. In fact, the toxicity of alkaloids could be reduced by their encapsulation in nanosystems (Table 1), which ensure a sustained release of the drug in therapeutic non-toxic concentrations, as well as an exclusive delivery solely to the infected cells.

### 2.1. Quinine

Quinine is a cinchona alkaloid extracted from the bark of *Cinchona officinalis* (*Rubiaceae*). Although it is the oldest antimalarial drug, it still remains the first line of treatment in most African countries. In addition, quinine has a critical role in the treatment of malaria in the first trimester of pregnancy [29]. The mechanism of its activity is related to its rapid schizonticidal effect against intra-erythrocytic malaria parasites. It is also gametocytocidal for *P. vivax* and *P. malariae*, but not for *P. falciparum*. However, the therapy with quinine has some limitations, in particular, a low therapeutic index, cardiotoxic side effects, and poor compliance with complex dosing regimens [30]. It was reported that the malaria parasites develop resistance to quinine due to low concentrations of quinine in the presence of a high parasitic load. Thus, the high intra-erythrocytic concentration of the drug could overcome the problem with the required high dose. In this view, the development of innovative drug delivery forms of quinine that would be able to optimize its efficacy would be highly advantageous. For instance, Haas et al. discovered that the encapsulation of quinine in poly(ɛ-caprolactone) nanocapsules (176 nm) increased the partition coefficient of the drug almost twice in infected erythrocytes (6.25 ± 0.25) compared to the pure drug (3.03 ± 0.07) due to an improved interaction between the nanosystem and the erythrocytes [9]. The latter resulted in stronger antimalarial effect after intravenous administration of the encapsulated quinine in *P. berghei*-infected rats. In particular, the administration of the encapsulated drug resulted in 100% survival at a lower dose (75 mg/kg/day) compared to the effective dose of the pure drug (105 mg/kg/day). Michels et al. [10] prepared polysorbate-coated Eudragit RS 100 nanospheres and nanocapsules aiming to improve the antimalarial effect of the drug in *P. berghei*-infected mice. The results revealed that quinine-loaded Eudragit nanocapsules increased survival time by about 6–7 days compared to the group treated with the free drug. The higher efficacy was explained by the improvement of the interaction between the membrane of the erythrocytes and the cationic nanocapsules. In addition, the coating with polysorbate 80 provided a repulsive steric barrier against opsonization from biological components. As a result, the free drug was detected after up to 8 h in the blood plasma, whereas the encapsulated quinine was detected for a longer time (24 h). Furthermore, the coated layer reduces the tension between the nanoparticles and the cells and contributes to a better interaction. Indeed, the incubation of the coated nanocapsules with infected erythrocytes showed a significantly higher partition coefficient of quinine compared to that of the free drug. In another study, quinine was loaded in Eudragit RS or poly(ɛ-caprolactone) nanocapsules simultaneously with curcuma oil [11]. Both types of nanocapsules showed lower levels of parasitemia and a high survival mean time (approximately 15 days) of *P. berghei*-infected mice. Mesoporous silica nanoparticles were also examined as carriers of quinine, taking into consideration their effective cellular uptake [31]. An additional functionalization or capping of their surface could enable the uptake or optimize the release rate, respectively. Amolegbe et al. loaded quinine in mesoporous silica nanoparticles MCM-41 and 3-phenylpropyl silane functionalized MCM-41 nanoparticles [12]. The non-functionalized MCM-41 particles exerted stronger antimalarial activity in *P. berghei* NK65-infected mice (ED_50_ < 0.0625 mg/kg) compared to the functionalized MCM-41 nanoparticles (ED_50_ 0.327 mg/kg) and the pure drug (15 mg/kg). A similar tendency was observed regarding the survival time of the infected mice.

As mentioned above, another advantage of nanosized drug delivery systems is the possibility to reduce the toxicity of the loaded drug by its gradual release and specific delivery to the infected cells. Izaguirry et al. reported that the side effects of quinine on the reproductive system could be reduced by encapsulation of the drug in nanocapsules [13]. The study observed that pure quinine decreased the spermatozoa membrane integrity and the follicular viability in male and female rats, respectively. For comparison, the nanoencapsulated quinine showed spermatozoa with 100% membrane integrity in male rats and 17β-hydroxysteroid dehydrogenase activity and follicular viability at control levels in female rats.

### 2.2. Piperine

Piperine, an alkaloid isolated from the seed of *Piper nigrum* (*Piperaceae*), has been reported as a probable antimalarial molecule [32]. It has been underlined that the main mechanism of the antimalarial effect is related to it acting on the erythrocytic phase, specifically, changing the morphology of the erythrocytes and rendering them defective. The advantageous properties of piperine are related to low toxicity and a low risk of resistance [32,33]. Oral administration of piperine (40 mg/kg) in curative and prophylactic assays suppressed parasitemia by 79.21% and 58.8%, respectively [34]. Some semisynthetic analogs of piperine could be even more efficient, e.g., 2,5-dimethoxy-substituted phenyl piperamide exerted a fivefold stronger effect against the 3D7 strain of *P. falciparum* than piperine [35]. Another strategy is a simultaneous administration of piperine with other antimalarial molecules. For example, in vitro methyl gallate and palmatine acted synergistically on hemozoin formation but did not exert antimalarial activity in vivo [36]. Interestingly, the administration of their combination with 25 mg/kg piperine in *P. berghei* NK65-infected mice reduced the parasitemia by more than 40%, suggesting a synergistic inhibition of hemozoin. A combination of curcumin and piperine was also examined in *P. berghei* ANKA-infected mice [37]. The study demonstrated a delayed onset of clinical symptoms and a significantly prolonged survival rate in the group treated with the combination (curcumin 300 mg/kg and piperine 20 mg/kg). Because of the low solubility of both molecules, the combination of piperine and curcumin was encapsulated in chitosan-alginate nanoparticles that were further included in microneedles for transdermal malaria treatment [14]. The developed microneedles effectively inhibited the *P. falciparum* FCR3 strain parasite (IC_50_ of 35.9 μg/mL), with no toxic and hemolytic effects.

### 2.3. Cryptolepine

Cryptolepine is an indoloquinoline alkaloid derived from *Cryptolepis sanguinolenta* (*Apocynaceae*). Its antimalarial effect in vitro against *P. falciparum* strains was reported in several studies [38,39,40]. For instance, its effect against *P. falciparum* strains K1 (multidrug-resistant strain) and T996 (chloroquine-sensitive clone) was comparable with that of chloroquine [38]. The main limitation for its clinical examination is its cytotoxicity as a result of intercalation into DNA and the inhibition of DNA synthesis. Kuntworbe and Al-Kassas [15] examined the possibility of reducing the hemolytic effect of cryptolepine by its loading in gelatin nanoparticles. Their results revealed that drug-loaded nanoparticles reduced the hemolytic tendency by almost four times compared to the non-encapsulated drug. Also, the sustained release of the drug (approximately 192 h) was considered an important prerequisite for effective antimalarial therapy because of the longer exposition of erythrocytes to the drug.

## 3. Sesquiterpene Lactones

Sesquiterpene lactones are a type of sesquiterpenoids that exert an antimalarial effect due to the generation of reactive radicals and the irreversible alkylation of heme and parasite proteins, leading to parasite death. The main agent from this group is artemisinin, although other molecules isolated from *Artemisia* species, such as ridentin and hanphyllin, were also active [41]. Some non-artemisinin agents were also examined. Parthenin derived from *Parthenium hysterophorus* (*Asteraceae*) and parthenolide derived from *Tanacetum parthenium* (*Asteraceae*) exhibited a similar effect on the *Plasmodium* blood stage development as that of artemisinin [42]. Unlike artemisinin, they have an effect on the transmissible stages of *P. falciparum*. Other sesquiterpene lactones (vernopicrin and vernomelitensin) extracted from *Vernonia guineensis* (*Asteraceae*) also demonstrated antiplasmodial activity against *P. falciparum* strains [43].

Artemisinin is a sesquiterpene lactone isolated from *Artemisia annua* (*Asteraceae*). It acts rapidly in the erythrocyte stage of the human host as a blood schizonticide. However, its poor water solubility, extensive first-pass metabolism and short half-life result in a low and variable oral bioavailability. In this view, Valissery et al. reported an increased water solubility of artemisinin by its encapsulation in poly(ε-caprolactone) nanoparticles or liposomes [6]. The authors found that the encapsulated artemisinin administered as an aqueous nanoparticle dispersion in *P. berghei*-infected mice exerted a comparable antimalarial effect to a referent solution of the pure drug. The results demonstrated the advantage of the nanoparticles, taking into consideration that the pure drug is not active at all as a water suspension.

Albumin nanoparticles are advantageous nanosystems, since they could enable specific targeting of the infected erythrocytes. In particular, it was reported that the malaria parasites import albumin in erythrocytes themselves [44]. In addition, albumin could allow for a high loading of artemisinin in nanoparticles because of the possibility for interaction through its tiol and amino groups. Based on these advantages, Ibrahim et al. [16] developed artemisinin-loaded albumin nanoparticles, suitable for intravenous administration. The results demonstrated that the encapsulated artemisinin had 100% bioavailability and exerted a strong effect in *P. falciparum*-infected mice. Thus, the formulation of artemisinin in albumin nanoparticles avoids the use of organic solvents or cosolvents for formulation of the drug in a dosage form.

Isacchi et al. loaded artemisinin in conventional and pegylated liposomes and its antimalarial effect was tested in *P. berghei* NK-65-infected mice [17]. Combined loading of artemisinin and curcumin in the liposomes was also performed. The pure artemisinin decreased parasitemia levels only 7 days after the start of administration and showed fluctuation in blood concentration. For comparison, all liposomal formulations exerted an immediate antimalarial effect and less variability in artemisinin plasma concentrations. The latter suggested that the modified release of drug(s) resulted in a constant antimalarial effect over time. However, the most significant effect was provided by the pegylated liposomal formulation, which was a consequence of the longer circulation in the blood, which enabled contact with the erythrocytes. Yaméogo et al. developed two types of surface-decorated nanoparticles based on γ-cyclodextrin (γ-CD), namely an mPEG-decorated/γ-CD nanoreservoir system and polysorbate 80/γ-CD nanospheres [18]. The circulation of the nanosystems was longer than that of the pure artemisinin after intravenous administration in healthy rats, allowing for sustained drug release in the blood. The plasma concentration of the pure drug was not detectable after 2 h, whereas the encapsulated artemisinin was still detectable after 6 h (nanoreservoirs) and 8 h (nanospheres). The mean plasma half-life of artemisinin from the nanospheres was 1.56-fold higher than that of the nanoreservoir. The other pharmacokinetic parameters of the nanospheres were also better compared to polysorbate 80/γ-CD- nanoreservoirs, suggesting the importance of morphology and surface modification on the biodistribution of the nanosystems.

## 4. Curcuminoids

Curcuminoids (curcumin, bisdemethoxycurcumin, demethoxycurcumin) are phenolic compounds that are extracted from the rhizomes of *Curcuma longa* (*Zingiberaceae*). The antimalarial effect of curcumin as well as its derivatives has been investigated [45,46,47,48]. It has been observed that the methoxyphenol group in curcumin’s structure is crucial for its antimalarial effect [49]. The antiplasmodial activity of curcumin is based on different mechanisms [50]. In particular, it inhibits the host glycogen synthase kinase-3 that provokes phosphorylation of NF-B, modulating the regulation of pro- and anti-inflammatory cytokine levels [51]. Curcumin induces the generation of ROS in parasite cells, further provoking protein and DNA damage within the cells. Furthermore, curcumin disrupts the transmission and development of *Plasmodium* parasites at the erythrocytic stage by inhibition of the formation of hemozoins [52]. However, the main limitations of its clinical implementation are its low solubility, rapid metabolism, and poor oral bioavailability. Various types of nanoparticles have been designed aiming to overcome the mentioned limitations. Lipid-based drug delivery nanosystems appear to be appropriate carriers because curcumin is more soluble in lipids, especially in liquid lipids. Nayak et al. formulated curcuminoids in nanostructured lipid carriers (diameter less than 250 nm), applying a mixture of solid and liquid lipids [4]. The structure of the system provided an initial burst release of curcuminoids, allowing for the exposure of the parasite to the increased serum drug concentration. In the second stage, a controlled release ensured prolonged exposure of the remaining parasites to the drug. Lipid nanosystems with a diameter 30–40 nm enabled intracellular transport in Caco-2 cells due to the increased solubility of curcumin [19]. The antimalarial effect in *P. berghei*-infected mice was modest after oral administration of the system. However, the combination with an encapsulated β-arteether in a subtherapeutic dose increased the survival rate of the mice. Rashidzadeh et al. loaded curcumin in lipid nanoparticles based on coconut oil and cetyl palmitate with a mean diameter of 145 nm [20]. The antiplasmodial activity of the formulation was examined in mice infected with *P. berghei*. The results showed significantly higher antimalarial activity of the encapsulated curcumin compared to that of the pure drug at a dose of 40 mg/kg/day.

Two types of liposomal formulations, Eudragit-hyaluronan liposomes and Eudragit-water-soluble dextrin (abbreviated as Eudragit-nutriosomes), were studied as curcumin delivery systems [21]. Oral administration of both types in *P. yoelii*-infected mice revealed that the curcumin into the Eudragit-nutriosomes enhanced the survival of all infected mice up to 11/11 days (at a dose 25/75 mg/kg) compared to 6/7 days for the treatment with the pure drug. The authors reported that the weaker effect of the Eudragit-hyaluronan liposomes was due to their low stability after reconstitution.

Another study applied curcumin-loaded poly(D,L-lactic-co-glycolic acid) nanoparticles (291 nm) at two concentrations (5 mg/kg and 10 mg/kg) in mice [22]. The administration of the lower concentration of the encapsulated curcumin resulted in a significantly higher suppression of the parasite (56.8%) compared to the free drug (40.5%). Akhtar et al. encapsulated curcumin in chitosan nanoparticles that demonstrated stronger inhibition of parasite lysate-induced heme polymerization in vitro than chloroquine [23]. Oral administration of the encapsulated curcumin in *P. yoelii*-infected mice cured all the mice, whereas with the pure drug, only one third of the mice survived. The improved activity of the encapsulated drug was explained with its higher stability in mouse plasma and the capacity of the nanoparticles to cross the mucosal barrier.

Some scientific groups have investigated different combinations of curcumin with other antimalarial molecules. Velasques et al. [24] performed simultaneous encapsulation of curcumin and quinine in polysorbate-coated polymeric nanocapsules (200 nm), aiming to overcome the limitation of quinine monotherapy. The results demonstrated significant reduction in *P. falciparum* parasitemia. Furthermore, the oral administration in *Caenorhabditis elegans* did not disrupt the reproductive capacity of the worms, which is a typical side effect of quinine. Curcumin and artesunate were loaded in poly(D,L-lactic-co-glycolic acid) nanoparticles with a mean diameter of 251 nm [25]. The antiplasmodial effect of the encapsulated combination was tested in Peters’ *P. berghei* suppressive test (5 and 10 mg/kg doses). The suppression of *P. berghei* was significantly stronger after the treatment with the nanoencapsulated combination compared to their non-encapsulated combination.

## 5. Flavonoids

Flavonoids are a large group of natural compounds possessing phenolic groups that are associated with their antioxidant, anti-inflammatory, and antitumor effects. The phenolic groups are also related to their antimalarial effect. These groups can be converted into phenoxy anions under in vivo cellular oxidative stress. The resulting anions provoke oxidative damage of parasite cellular components or direct tissue damage through interactions with structural proteins or DNA of the parasites [53]. The opportunity to combine the antiplasmodial effect of flavonoids with their anti-inflammatory and antioxidant activity makes them attractive for antimalarial therapy [54,55].

### 5.1. Quercetin

Quercetin is a flavonol whose mechanisms of antimalarial activity are related to the inhibition of the hemozoin formation, reduction in the levels of pro-inflammatory cytokines, and augmentation of the levels of anti-inflammatory cytokines [56,57]. Quercetin is a hydrophobic molecule characterized by poor absorption, extensive metabolism, and low oral bioavailability. Therefore, its encapsulation in nanosized drug delivery systems could significantly enhance its administration as well as its effects. For instance, phytosomes loaded with quercetin showed 92.31% anti-parasitic activity against *P. falciparum* at 400 µg/mL concentration in comparison with the free drug (78.87% at the same concentration) [26]. Moreover, the system was non-toxic in healthy lung fibroblast WI38 cells and showed no hemolytic activity, making it appropriate as an adjuvant antimalarial drug. Davoodi et al. also prepared quercetin phytosomes, which in combination with hydroxychloroquine sulfate showed promising alleviation of malaria adverse effects regarding liver damage and inflammation in *P. berghei*-infected mice [58]. Fulgheri et al. developed nanovesicle-doped nanoemulsions loaded with quercetin alone or with a combination of quercetin and artemisinin [27]. The co-loaded system inhibited in vitro the growth of *P. falciparum* (3D7 strain) with IC_50_ of 0.009 µg/mL. The IC_50_ of non-loaded and single-loaded quercetin was 4.3 and 1 µg/mL, respectively, while for artemisinin, the values were 0.019 and 0.018 µg/mL. Thus, the encapsulation of quercetin even alone enhanced its in vitro antimalarial activity. Further, the double-loaded nanoemulsion led to significant improvement of the survival rate of *P. yoelii* 17XL-infected mice compared to the animals treated only with artemisinin solution. Most importantly, 33% of the mice treated with the co-loaded system completely recovered after clearing parasitemia below detectable levels and developed immunity to malaria. Low toxicity (<15%) was observed in human colon adenocarcinoma (Caco-2) and human umbilical vein endothelial (HUVEC) cells in 2.5, 5, 10, and 20 μg/mL concentrations, assuming safe application. The authors also observed that the co-loaded nanoemulsion was stable when diluted at gastrointestinal pH and high ionic strength, making it suitable for oral administration.

### 5.2. Luteolin

Luteolin is a flavonoid that exerts antimalarial activity by the inhibition of three important enzymes (FabG, FabZ, and FabI) involved in the fatty acid biosynthesis of *P. falciparum* [59]. The synthesis of fatty acid in parasites is an important stage of the formation of new organelles and biomembranes. Furthermore, luteolin showed in vitro activity against chloroquine-sensitive and chloroquine-resistant strains of *P. falciparum* in the low to submicromolar range. Another study reported that luteolin inhibited the progression of the growth of the parasite beyond the young trophozoite stage at concentrations 11–12 µM [60]. Thus, the low toxicity of luteolin and its antimalarial activity are a prerequisite for its clinical administration, but its low solubility results in poor bioavailability. In this view, Puttappa et al. hypothesized that luteolin or quercetin could be co-loaded in a self-nano-emulsifying drug delivery system along with artemisinin, aiming to fight resistant *P. falciparum* parasites [61]. These systems would enhance the absorption and bioavailability of the active agents, increase their half-life, decrease their toxicity, and possibly reduce first-pass metabolism. Despite the potential of luteolin for treating malaria, there are still no experimental studies on the effect of its nanoencapsulated form.

## 6. Antibiotics

The administration of antibiotics combining antiprotozoal and antibacterial activity appears to be an advantageous therapy. The existing studies have mainly reported the effect of some polyether ionophores like monensin and nigericin [62,63,64] and some newly isolated agents [65].

Monensin is a polyether antibiotic isolated from *Streptomyces cinnamonensis*. It has been reported that the mechanism of its antimalarial activity is related to the alkalinization of the food vacuoles of the parasite (as an ionophore it carries Na^+^ across the membranes and elevates Na^+^ concentration in the parasite vacuoles) and the induction of eryptosis [62,64]. However, the strong hydrophobic properties of monensin make its formulation in dosage forms difficult and hinder its clinical administration. Thus, incorporation in appropriate nanosized systems, e.g., formulated with lipid carriers, could avoid the use of toxic solvents. For instance, monensin was loaded in PEGylated liposomes containing stearylamine (SA) or phosphatidic acid (PA), and the effect of the formulations was studied on *P. berghei* NK65-infected mice [28]. The experiment showed that the effect depended on the lipid composition and density of the PEG-layer. The most effective were the liposomes formulated with the positively charged lipid stearylamine (SA) and the highest PEG 2000 density (5 mol%), followed by liposomes with the same PEG density but without stearylamine. In particular, the survival time of the mice treated with the PEGylated SA liposomes was 27.5 days; with the PEGylated liposomes, 23.5 days; and with the PEGylated PA liposomes, 22 days. It seemed that the higher density of PEG 2000 (this chain length was optimal compared to 750, 1000, 3000, and 5000) provided the required long circulation and more effective contact with erythrocytes. On the other hand, the cationic lipid enabled electrostatic interaction with the negatively charged membrane of the infected erythrocytes that had increased the levels of phosphatidylserine. This interaction may induce a disruption of their membrane and cell death. In all cases, the administration of the liposomes resulted in a higher efficacy compared to that of the pure monensin. Furthermore, the study reported that the liposomal formulations were preferentially internalized by the infected erythrocytes due to a decrease in the surface pressure and promotion of the cellular uptake.

## 7. Metal Nanoparticles Obtained by Green Synthesis from Plant Extracts

Green synthesis of metal nanoparticles is a simple, cost-effective, and economical approach for the preparation of metal nanoparticles using plant extracts. The method is eco-friendly since toxic chemicals are not used. Regarding malaria treatment, the main advantage of these nanoformulations is that the plant extracts offer a variety of phytocompounds (e.g., compounds with anti-inflammatory and/or antioxidant activity) that would have a further beneficial effect on malaria treatment. The limitation of this strategy is related to safety problems such as the potential toxicity of metal nanoparticles on cells, tissue, and organs. Green synthesis is most often applied for the production of silver, gold, and zinc oxide nanoparticles (Table 2), which are discussed in the followed subsections.

### 7.1. Silver Nanoparticles (AgNP)

Silver nanoparticles are typically obtained by reducing a silver nitrate (AgNO_3_) using various biomolecules in plant extracts (e.g., enzymes, proteins, polysaccharides, alkaloids, saponins, tannins, amino acids, vitamins, etc.). The main advantage of silver nanoparticles is that they combine antiprotozoal and antibacterial activity [5]. Their antimalarial effect is related to the release of silver ions that are toxic to parasites, including *Plasmodium*. The released ions disturb the integrity and fluidity of the cell membrane of the parasite, causing apoptosis or necrosis [66]. In addition, silver nanoparticles induce oxidative stress and damage of cellular components through the generation of reactive oxygen species. The limitations of silver nanoparticles are related to the reactivity of silver ions, stability, and toxicity. However, their strong antiprotozoal and antibacterial effect, as well as the possibility to reduce their toxicity, makes their implementation attractive in malaria treatment. Al-Quraishy et al. obtained silver nanoparticles from *Indigofera oblongifolia* extract using green synthesis [66]. In fact, a hepatoprotective effect of the leaf extract from this plant against *P. chabaudi*-induced hepatic injury in mice has been previously observed [67]. The developed silver nanoparticles suppressed the parasitemia in *P. chabaudi*-infected mice spleen to approximately 98% (7 days postinfection) [66]. The silver nanoparticles restored the levels of glutathione, nitric oxide, and catalase to values which were close to those of an uninfected spleen. Furthermore, the silver nanoparticles downregulated the expression of IL-1b, IL-10, and TNF-α, which were altered by the infection. Thus, the nanoparticles functioned as an antioxidant and anti-inflammatory agent, probably due to the other phytocompounds of the extract used for their preparation.

A recent study reported on a combination of the antimicrobial properties of silver nanoparticles and the antimalarial activity of the compounds in the extract used for their production. The silver nanoparticles were prepared by green synthesis using a crude extract from *Sargassum tenerrimum* (brown marine seaweed) [68]. The results from in vitro studies showed antiplasmodial activity of the nanoparticles with IC_50_ values of 7.71 µg/mL and 23.93 µg/mL against *P. falciparum* and *P. berghei*, respectively. In an in vivo study, the nanoparticles significantly reduced the parasitemia in *P. berghei*-infected mice without adverse toxic effects. The authors concluded that the combination of the antimicrobial properties of silver and the antimalarial properties of *Sargassum tenerrimum* metabolites (e.g., meroterpenoids, phlorotanins sterols, glycolipids, and fucoidans) is an alternative for therapeutic application. Similar silver nanoparticles (32 nm) combining antibacterial and antimalarial activity were synthesized using aqueous extracts of the leaf and fruit of *Crataegus ambigua* [69]. An in vitro assay revealed that the plant extract alone exerted a moderate inhibition (48.5%) of *P. falciparum* (NF54 strain), whereas the nanoparticles produced 100% inhibition. The result emphasized the benefit of the phytocompounds of the extract on the overall activity of the prepared nanoparticles. At the same concentration (20 µg/mL), the inhibition of the parasite upon chloroquine treatment was equal to that of the developed silver nanoparticles. Moreover, the nanoparticles possessed a strong antibacterial effect on seven bacterial strains, which could be advantageous for therapeutic purposes.

### 7.2. Gold Nanoparticles (AuNP)

Gold nanoparticles could be applied in the therapy of malaria considering the studies on the antiprotozoal activity of gold complexes [70]. The authors observed an antimalarial effect of the gold-based antiarthritic drug auranofin in the same study [70]. It was postulated that the reactivity of the gold toward thiol and selenol groups of proteins led to the direct inhibition of *P. falciparum* thioredoxin reductase. The inhibition of the enzyme causes severe intracellular oxidative stress, to which *P. falciparum* is highly sensitive. Another study discovered that glucose-based gold nanoparticles targeted all asexual and sexual erythrocytic stages of the parasite without unspecific binding or lysing of the erythrocytes [71]. The authors concluded that the gold nanoparticles probably interacted with the cysteine-rich proteins on the surface of *P. falciparum* that mediate the invasion of the parasite in the human erythrocytes.

Considering the rich phenolic content and strong antioxidant effect of *Syzygium jambos* (*Myrtaceae*), Dutta et al. synthesized gold and silver nanoparticles (diameter less than 10 nm) using aqueous extract of leaves and bark [72]. An in vitro antiplasmodial assay on the chloroquine-sensitive (3D7) and resistant (Dd2) strains of *P. falciparum* revealed that silver nanoparticles exerted a stronger effect compared to the gold nanoparticles and the plant extracts. This research proved the concept of the benefit of the phytocompounds from the plant extract on the biological action of metal particles obtained through green synthesis. The study compared the developed gold and silver nanoparticles with their counterparts obtained through chemical synthesis, which possessed a weaker antiplasmodial effect and higher cytotoxicity on rat skeletal cells.

Gold nanoparticles were also prepared using the aqueous extract of leaves of *Coccinia grandis*. In *P. berghei* NK-65-infected mice, the biosynthesized gold nanoparticles showed suppression of the parasite by 88.75%, which was higher than the aqueous extracts of the plant (69.45%) [73].

### 7.3. Zinc Oxide Nanoparticles (ZnONP)

ZnO nanoparticles were synthesized with the help of aqueous peel extract of *Lagenaria siceraria* [74]. The nanoparticles were tested against III-instar larvae of *Anopheles stephensi,* as well as against the *P. falciparum* chloroquine-sensitive strains. In a beta-hematin formation assay, the biosynthesized ZnO nanoparticles demonstrated higher antimalarial activity (IC_50_ = 1.38 mg/mL) compared to chloroquine (IC_50_ = 0.91 mg/mL) [74]. Another study reported green synthesis of ZnO nanoparticles using *Rhazya stricta* leaf extract [75]. The nanoparticles were discovered to be effective, with an IC_50_ value of 3.41 μg/mL. The synthesized nanoparticles had minimum hemolytic activity (4.43%) at maximum concentration and showed better biocompatibility.

**Table 2:** Examples for green-synthesized metal nanoparticles intended for malaria treatment.

Nanoparticle Type	Source	Achievement	Reference
AgNP	*Azadirachta indica* (*Meliaceae*)	Stronger antiplasmodial effect of AgNP compared to the plant extracts or amylase alone	[76]
	*Saraca asoca* (*Fabaceae*)		
	Purified Alpha Amylase		
AgNP	*Indigofera oblongifolia* (*Fabaceae*)	Suppression of the parasitemia in *P. chabaudi*-infected mice to approximately 98%	[66]
		Downregulation of the expression of IL-1b, IL-10 and TNF-α	
AgNP	*Sargassum tenerrimum* (*Sargassaceae*)	Antiplasmodial activity against *P. falciparum* and *P. berghei*	[68]
		Significant reduction in the parasitemia in *P. berghei*-infected mice without adverse toxic effects	
AgNP	*Crataegus ambigua* (*Rosaceae*)	Equal inhibition of *P. falciparum* (NF54 strain) with that of chloroquine	[69]
		Strong antibacterial effect against seven bacterial strains	
AgNP	*Murraya koenigii* (*Rutaceae*)	Antiplasmodial activity on chloroquine-sensitive *P. falciparum* (3D7)	[77]
AgNP	*Azadirachta indica* (Meliaceae)	Antiplasmodial activity against *P. falciparum* (3D7 and RKL9 strains)	[78]
AgNP	*Euphorbia cotinifolia* (*Euphorbiaceae*)	ROS production and disruption of the redox equilibrium of parasite Apoptosis of *P. falciparum*-infected erythrocytes	[79]
AgNP	*Syzygium jambos* (*Myrtaceae*)	Stronger in vitro antiplasmodial effect of AgNP compared to AuNP	[72]
AuNP			
AuNP	*Coccinia grandis* (*Cucurbitaceae*)	Suppression of parasite by 88.75%	[73]
ZnONP	*Lagenaria siceraria* (*Cucurbitaceae*)	Antimalarial activity comparable to chloroquine	[74]
ZnONP	*Rhazya stricta* (*Apocynaceae*)	Antiplasmodial effect (IC50 of 3.41 μg/mL)	[75]
		Minimum hemolytic activity	

## 8. Perspectives

Innovative drug delivery systems like nanoparticles could solve the limitations of the traditionally established drugs in malaria therapies. Nanoparticles could decrease premature drug elimination and ensure the long circulation and exposure of the infected erythrocytes to the loaded drug. In addition, they are able to provide site-specific delivery exclusively to the *Plasmodium*-infected erythrocytes. Because of the complexity of the life cycle of the parasite, future development must be related to the formulation of decorated nanoparticles that act through receptors on the host cells or malaria parasites. In addition to surface decoration, it could be advantageous if the carriers of the nanoparticles exert antimalarial effects themselves, e.g., silver nanoparticles have demonstrated activity against both the parasite and the vector. In this view, an important strategy is the development of silver nanoparticles prepared by green synthesis from various plant extracts, including those containing antimalarial compounds, like the *Artemisia* species. The simultaneous encapsulation of combinations of antimalarial drugs is also a potential approach. Thus, the nanosized drug delivery systems are a powerful strategy because of the opportunity to improve specificity of the therapy as well as to reduce the drug dosage frequency, treatment time, and toxicity.

## Figures and Tables

**Table 1:** Examples for natural substances encapsulated in nanoparticles intended for malaria treatment.

Natural Product	Nanocarrier	Achievement	Reference
Quinine	Poly(ɛ-caprolactone) nanocapsules	Increased intra-erythrocytic concentration	[9]
	Polysorbate coated Eudragit RS 100 nanospheres and nanocapsules	Improved interaction between the drug and erythrocytic membrane	[10]
	Eudragit RS or poly(ɛ-caprolactone) nanocapsules (co-loaded with curcuma oil)	Decreased level of parasitemia and increased survival rate	[11]
	Mesoporous silica nanoparticles	Increased antimalarial activity and survival rate due to increased uptake	[12]
	Polysorbate-coated nanocapsules	Decreased toxicity on reproductive system	[13]
Piperine	Chitosan-alginate nanoparticles (co-loaded with curcumin)	Enhanced antimalarial activity against *P. falciparum* No toxicity and hemolytic activity	[14]
Cryptolepine	Gelatine nanoparticles	Reduced hemolysis and longer exposition of the drug to erythrocytes	[15]
Artemisinin	Poly(ε-caprolactone) nanoparticles and liposomes	Increased aqueous solubility	[6]
	Albumin nanoparticles	Increased solubility and bioavailability Parasite targeting and strong antimalarial effect	[16]
	Pegylated liposomes (alone or co-loaded with curcumin)	Less variability in plasma concentrations Longer blood circulation and contact with erythrocytes	[17]
	mPEG-decorated/γ-CD nanoreservoir system and polysorbate 80/γ-CD nanospheres	Longer blood circulation and higher mean plasma half-life	[18]
Curcumin	Nanostructured lipid carriers	Controlled release, resulting in prolonged exposure of the parasites to the drug	[4]
	Lipid nanoparticles (alone or co-loaded with subtherapeutic dose β-arteether)	Increased solubility and survival rate	[19]
	Lipid nanoparticles	Higher antimalarial activity against *P. berghei*	[20]
	Liposomes—Eudragit-hyaluronan liposomes and Eudragit-water-soluble dextrin	Increased survival rate	[21]
	Poly(D,L-lactic-co-glycolic acid) nanoparticles	Increased suppression of malarial parasite	[22]
	Chitosan nanoparticles	Higher stability and capacity of crossing the mucosal barrier Stronger activity in vivo against *P. yoelii*	[23]
	Polysorbate-coated polymeric nanocapsules (co-loaded with quinine)	Reduction in *P. falciparum* parasitemia Decreased toxicity on reproductive system of *Caenorhabditis elegans*	[24]
	Poly(D,L-lactic-co-glycolic acid) nanoparticles (co-loaded with artesunate)	Increased suppression of *P. berghei* compared to non-loaded combination of chloroquine and artesunate	[25]
Quercetin	Phytosomes	Higher activity against *P. falciparum* compared to pure quercetin Absence of in vitro toxicity and hemolytic activity	[26]
	Nanovesicle-doped nanoemulsions (quercetin alone or co-loaded with artemisinin)	Enhanced antimalarial effect on *P. falciparum*; Increased survival rate (co-loaded system); Low in vitro toxicity	[27]
Monensin	PEGylated liposomes	Long blood circulation Increased interactions with erythrocytic membranes Preferential internalization in erythrocytes	[28]

## Data Availability

All data are contained within the article.
